# Determinants of the Proinflammatory Action of Ambient Particulate Matter in Immortalized Murine Macrophages

**DOI:** 10.1289/ehp.1002105

**Published:** 2010-07-27

**Authors:** Cecilia Guastadisegni, Frank J. Kelly, Flemming R. Cassee, Miriam E. Gerlofs-Nijland, Nicole A.H. Janssen, Roberta Pozzi, Bert Brunekreef, Thomas Sandström, Ian Mudway

**Affiliations:** 1 Department of Environment and Primary Prevention, Instituto Superiore di Sanita, Rome, Italy;; 2 MRC-HPA Centre for Environment and Health, King’s College London, London, United Kingdom;; 3 Centre for Environmental Health, National Institute for Public Health and the Environment, Bilthoven, the Netherlands;; 4 Institute for Risk Assessment Sciences, Utrecht University, Utrecht, the Netherlands;; 5 Department of Technology and Health, Instituto Superiore di Sanita, Rome, Italy;; 6 Department of Respiratory Medicine and Allergy, Umeå University, Umeå, Sweden

**Keywords:** copper, endotoxin, inflammation, iron, macrophages, metals, particulate matter, polyaromatic hydrocarbons

## Abstract

**Background:**

Proximity to traffic-related pollution has been associated with poor respiratory health in adults and children.

**Objectives:**

We wished to test the hypothesis that particulate matter (PM) from high-traffic sites would display an enhanced capacity to elicit inflammation.

**Methods:**

We examined the inflammatory potential of coarse [2.5–10 μm in aerodynamic diameter (PM_2.5–10_)] and fine [0.1–2.5 μm in aerodynamic diameter (PM_0.1–2.5_)] PM collected from nine sites throughout Europe with contrasting traffic contributions. We incubated murine monocytic-macrophagic RAW264.7 cells with PM samples from these sites (20 or 60 μg/cm^2^) and quantified their capacity to stimulate the release of arachidonic acid (AA) or the production of interleukin-6 and tumor necrosis factor-α (TNFα) as measures of their inflammatory potential. Responses were then related to PM composition: metals, hydrocarbons, anions/cations, and endotoxin content.

**Results:**

Inflammatory responses to ambient PM varied markedly on an equal mass basis, with PM_2.5–10_ displaying the largest signals and contrasts among sites. Notably, we found no evidence of enhanced inflammatory potential at high-traffic sites and observed some of the largest responses at sites distant from traffic. Correlation analyses indicated that much of the sample-to-sample contrast in the proinflammatory response was related to the content of endotoxin and transition metals (especially iron and copper) in PM_2.5–10_. Use of the metal chelator diethylene triamine pentaacetic acid inhibited AA release, whereas recombinant endotoxin-neutralizing protein partially inhibited TNFα production, demonstrating that different PM components triggered inflammatory responses through separate pathways.

**Conclusions:**

We found no evidence that PM collected from sites in close proximity to traffic sources displayed enhanced proinflammatory activity in RAW264.7 cells.

Epidemiological studies have consistently demonstrated associations of acute and chronic exposures to airborne particulate matter (PM) with cardiopulmonary morbidity and mortality (Pope and Dockery 2006). Although this observation is robust across numerous studies, there is considerable heterogeneity in the size of PM effect estimates (Dominici et al. 2006). Because of a lack of data regarding the contribution of PM from different sources to human health outcomes, PM is often handled as a uniform pollutant defined by its aerodynamic diameter and ambient mass concentration, an imperfect surrogate of its biological activity (Borm et al. 2007). Evidence that composition and hence source are determinants of PM health effects comes from studies demonstrating increased respiratory symptoms in populations living in close proximity to busy roads. Exposure to traffic-related pollution has been associated with retarded lung development (Gauderman et al. 2007) and enhanced allergic and respiratory symptoms in children attending schools (Janssen et al. 2003) or living (Morgenstern et al. 2008) in close proximity to heavily trafficked roads.

The underlying basis for many of the observed health effects, including exacerbation of asthma and chronic obstructive pulmonary disease, may be related to the capacity of inhaled PM to induce airway inflammation (Brunekreef and Holgate 2002). PM-induced inflammation has been attributed to a range of factors, including *a*) its physicochemical characteristics (Brown et al. 2001); *b*) its capacity to elicit oxidative stress through the introduction of redox catalysts into the lung, including redox-active metals [e.g., iron (Fe), copper (Cu), nickel (Ni), vanadium (V)] and quinones (Ayres et al. 2008); and *c*) its capacity to trigger innate immune responses by acting as vectors for bacterial endotoxin or lipopolysaccharide (LPS) (Monn and Becker 1999; Osornio-Vargas et al. 2003; Schins et al. 2004). The metabolism of polyaromatic hydrocarbons (PAHs) by airway cells has also been shown to increase generation of intracellular reactive oxygen species (ROS), which can stimulate the release of inflammatory mediators (Baulig et al. 2003). A better understanding of the relative contribution of PM components to inflammatory potency would permit a more targeted approach to PM regulation focused on specific toxic components rather than the whole mass concentration (Grahame and Schlesinger 2007; Nel 2005).

In the present study, we tested the hypothesis that the cellular inflammatory response elicited by ambient PM is a function not only of ambient mass concentration but also of physicochemical characteristics that reflect different source contributions. Specifically, we investigated whether coarse [2.5–10 μm in aerodynamic diameter (PM_2.5–10_)] and fine [0.1–2.5 μm in aerodynamic diameter (PM_0.1–2.5_)] PM fractions collected from nine sites across Europe with contrasting traffic contributions and pollutant sources have differential effects on the stimulated release of proinflammatory mediators [interleukin-6 (IL-6), tumor necrosis factor-α (TNFα), and arachidonic acid (AA)] from the macrophage-like cell line RAW264.7. We assessed responses in relation to the compositional profile of the PM samples, and we performed inhibitory studies using the metal chelator diethylene triamine pentaacetic acid (DTPA) and recombinant endotoxin-neutralizing protein (rENP) to establish the contribution of PM metals and endotoxin to the inflammatory responses observed.

## Materials and Methods

### Cell lines and chemicals

The RAW264.7 mouse monocyte/macrophage cell line was kindly donated by B. Brune (University of Erlangen-Nurnberg, Erlangen, Germany) and maintained at the Istituto Superiore di Sanita as described previously (Guastadisegni et al. 1997). See Supplemental Material (doi:10.1289/ehp.1002105) for additional details of the cell line and chemicals.

### Environmental PM collection and chemical characterization

Parallel PM_2.5–10_ and PM_0.1–2.5_ samples were collected onto polyurethane foam using a high-volume cascade impactor (Bloemen et al. 2005; Janssen et al. 2008) at five sites in the Netherlands with differing traffic contrasts. Sites were classified as high, moderate, or low based on their proximity to major roads and the level of traffic: Amsterdam (AMS; high), 50 m from a highway with high car and truck traffic; Dordrecht (DOR; moderate), 300 m from a highway with moderate car and high truck traffic; Badhoevedorp (BAD; moderate), 200 m from a highway with moderate traffic; Sassenheim (SAS; low), 375 m from a highway with low to moderate traffic; and at the exit of the Hendrik-Ido-Ambacht (HIA; high), a freeway tunnel carrying a high number of diesel vehicles. Sampling was also performed at two sites in Munich, Germany: the Central Munich Ost Bahnhof (MOB; high), alongside a major four-lane highway and 40 m away from another busy highway; and at the suburban Munich Grosshadern Hospital (MGH; low), away from major roads. Details about all sites, other than HIA, have been described previously (Janssen et al. 2008). To increase variation in source composition, we included two more sites—Rome, Italy (ROM; high traffic), and Lycksele, northern Sweden (LYC; low traffic)—where PM was largely derived from domestic wood burning. All sites were visited at least twice (14 days per visit) over a 12-month period, with eight visits at the two Munich sites. A total of 41 samples were obtained, extracted, and chemically characterized before being tested for cellular toxicity.

Sample characterization was performed at the National Institute for Public Health and the Environment (RIVM), in the Netherlands, as previously described (Bloemen et al. 2005). Particle extraction from the foam substrates into methanol was achieved using a vortexing and sonication protocol as described previously by Cassee et al. (2003). We analyzed elemental composition of the particles using inductively coupled plasma mass spectrometry after sample digestion in dilute aqua regia. Secondary aerosol contributions were quantified with ion chromatography [chloride (Cl^−^), nitrate (NO_3_), and sulfate (SO_4_)] or photometry [ammonium (NH_4_)], with anions analyzed using a Dionex guard column (AG-4A), separation column (Dionex-AS-A4), and pulsed electrochemical detection (Dionex-PED; Dionex Benelux B.V., Amsterdam, the Netherlands). PAHs were analyzed on a 30 m × 0.25 mm WCOT DB-5MS column in a Fison 8000 series gas chromatograph (Fisons Instruments, Breda, the Netherlands) equipped with an Interscience MD800 mass spectrometer with electron impact and selected ion recording mode (Interscience BV, Breda, the Netherlands). For endotoxin analysis, approximately 2 mg PM was suspended in 1.2 mL pyrogen-free water with 0.05% Tween-20 and vortexed for 40 min. Endotoxin was measured in the extract using the kinetic *Limulus* mebocyte lysate test, as described by Douwes et al. (1995). Extracts were tested at a 5-fold dilution and quantified against a known endotoxin standard (BioWhittaker, Walkersville, MD, USA).

### Assessment of PM-induced proinflammatory responses and cell viability

We maintained the mouse monocytic-macrophagic cell line RAW264.7 in RPMI 1640 medium (HyClone, Cramlington, UK) supplemented with 100 U/mL penicillin, 100 μg/mL streptomycin, 10% heat-inactivated fetal bovine serum, and 2 mM Glutamax I (Life Technologies, San Giuliano Milanese, Italy) (complete RPMI 1640 medium). All experiments were performed using RPMI with 1% heat- inactivated fetal bovine serum. RAW264.7 cells were plated at a density of 1.3 × 10^5^ cells/well in 96-well plates. Cell cultures were allowed to adhere overnight and were stimulated with the particles the next day. Particles were resuspended in 1% serum culture medium, and cells were treated with particle concentrations at 20 and 60 μg/cm^2^ after 10 min of sonication (Bath Sonicator 5200; Branson Ultrasonics, Danbury, CT, USA). These concentrations have been shown to induce minimal cytotoxicity in RAW264.7 cells (Pozzi et al. 2005). After a 5-hr incubation, supernatants were collected, centrifuged at 2,000 rpm for 5 min, and frozen at −80°C before quantification of IL-6 and TNFα concentrations. All incubations were performed in duplicate in two or three separate experiments. TNFα was measured in nine samples to validate its use in the endotoxin inhibitor study. IL-6 and TNFα concentrations were determined using commercially available enzyme-linked immunosorbent assay (ELISA) kits (Biotrak cellular communication assays; Amersham Pharmacia Biotech, Uppsala, Sweden) according to the manufacturer’s instructions. Cell viability and membrane integrity were determined using an enzymatic colorimetric assay (Cytotoxicity Detection Kit; Roche Diagnostics, Ingelheim, Germany) to measure lactate dehydrogenase (LDH) release in the culture supernatants immediately after the 5-hr incubation period and in the cell lysates after a 20-min incubation with 1% Triton X-100 in phosphate-buffered saline. Results are expressed as LDH activity relative to controls (culture supernatant LDH minus control cell LDH) as a percentage of total LDH activity in cell lysate.

For AA determination, cells were prelabeled for 20–22 hr in complete media supplemented with 0.5 μCi/mL [^3^H]AA, washed three times with complete media, and then treated with test particles at concentrations of 20 or 60 μg/cm^2^. Aliquots of supernatants were collected after 5 hr and counted for radioactivity. The incorporation pattern of [^3^H]AA in RAW264.7 cells and the identification of [^3^H]AA in the cell supernatants have been described previously (Guastadisegni et al. 1997). Prostaglandin production in stimulated RAW264.7 cells is maximal after 24 hr of LPS stimulation (Guastadisegni et al. 1997), so it is likely that radioactivity measured after 5 hr of incubation largely reflects [^3^H]AA release.

We determined spontaneous basal release of IL-6, AA, and TNFα using an optimal concentration of LPS as a positive control run in parallel. Under these experimental conditions, the maximal LPS effect on RAW264.7 cells was observed at a concentration of 1 μg/mL.

### Inhibitor studies

Inhibition studies were performed using DTPA and rENP to ascertain the contribution of metals and endotoxin in ambient PM to inflammatory responses. We analyzed a panel of four samples from the original screening exercise reflecting the range of the observed responsiveness and from four different sites. Samples were preincubated at room temperature with 0.1 mM and 1.0 mM DTPA for 1 hr before addition to the RAW264.7 cells as described above; AA release was quantified after the 5-hr particle challenge. Preliminary experiments using 2–8 μg/mL rENP demonstrated that 2 μg/mL was optimal for reducing the endotoxin content of the selected PM samples (89 ± 6.3%). Therefore, we added 2 μg/mL rENP to PM suspensions immediately before their addition to the cell cultures. The impact of this treatment on TNFα release from cell cultures was assessed 5-hr postchallenge as described above.

### Statistical analyses

We assessed differences between sampling locations and between sites classified by traffic contribution using univalent analysis of variance with post hoc analysis with the Games-Howell test, assuming unequal variances and group sizes. Significant differences between sites were assumed at the 5% level. Associations between inflammatory mediators (AA and IL-6) and PM components were assessed using Pearson’s correlations. Correlation analysis was performed on all samples for each PM size fraction (*n* = 40–41) and using the means from each site (*n* = 9). We used SPSS (version 16; SPSS Inc., Chicago, IL, USA) to perform all statistical analyses.

## Results

### Cell viability

We quantified LDH leakage into the supernatant to confirm that the concentrations of coarse and fine PM did not induce cytotoxicity or altered membrane permeability. LDH release was limited (< 1% of the cellular content in the vast majority of cases) with no evidence of significant differences in cellular injury with PM from any of the sites or between the coarse and fine fractions [see Supplemental Material, Figure 1 (doi:10.1289/ehp.1002105)].

### AA release

**Figure 1:**
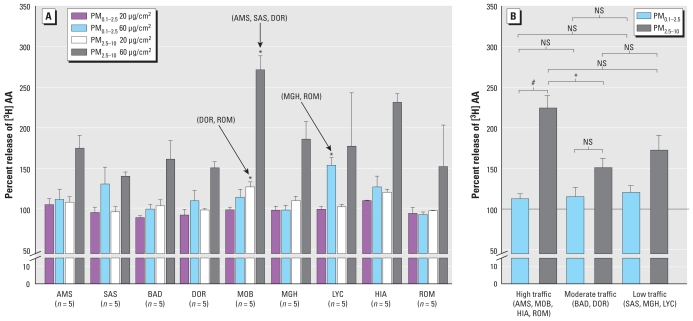
Release of [3H]AA from prelabeled RAW264.7 cells incubated for 5 hr with 20 or 60 μg/cm^2^ parallel PM_0.1–2.5_ and PM_2.5–10_ samples collected from nine European sites. AA release is expressed as a percentage relative to that observed from untreated cells. Data (site mean ± SE; *n* = 2–8 PM samples) are presented separately for each site (*A*) and by traffic classification (*B*). In *A,* site-specific contrasts were identified by univalent analysis of variance at a dose of 60 μg/cm^2^ for both PM_0.1–2.5_ (*p* = 0.049) and PM_2.5–10_ (*p* = 0.017). Post hoc analysis revealed significant differences between the sites indicated by the arrows with those summarized by their site code in parentheses. In *B*, significant differences between sites are classified by traffic and fraction. NS, a nonsignificant contrast. *p < 0.05, and ^#^*p* < 0.001.

Relative to particle-free controls, AA release did not significantly increase after exposure to any of the PM_0.1–2.5_ samples, with the exception of samples from LYC (mean ± SE, 147.0 ± 28.1% at 60 μg/cm^2^) (Figure 1A). In contrast, all PM_2.5–10_ samples significantly increased AA release relative to controls at 60 μg/cm^2^. AA release was significantly increased with exposure to PM_2.5–10_ samples from MOB (high traffic) compared with AMS (high traffic), SAS (low traffic), and DOR (moderate traffic) samples at 60 μg/cm^2^ and DOR and ROM (high traffic) samples at 20 μg/cm^2^. The only significant site differences for PM_0.1–2.5_ were for LYC (low traffic, wood burning) with MGH (low traffic) and ROM (high traffic) at 60 μg/cm^2^. When data were grouped by traffic characteristic and PM fraction, only the later significantly modified AA release (*p* < 0.001) in response to exposure, with coarse PM samples eliciting a greater response than fine PM samples from high-traffic sites (Figure 1B). We also observed differences in responses to coarse and fine PM for samples from moderate- and low-traffic sites, but contrasts were not statistically significant.

### IL-6 production

**Figure 2:**
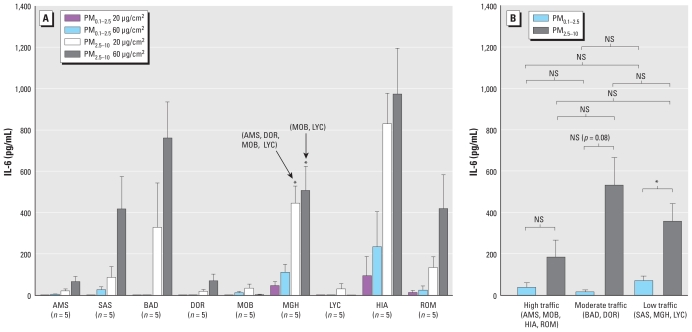
IL-6 production from RAW264.7 cells incubated with 20 and 60 μg/cm^2^ ambient PM_0.1–2.5_ or PM_2.5–10_ for 5 hr. Data (site mean ± SE; *n* = 2–8 PM samples) are presented separately for each site (*A*) and by traffic classification (*B*). In *A*, site-specific contrasts were identified by univalent analysis of variance at both doses of PM_0.1–2.5_ (*p* = 0.038 for 20 μg/cm^2^; *p* = 0.005 for 60 μg/cm^2^) and PM_2.5–10_ (*p* = 0.001 for 20 μg/cm^2^; and *p* = 0.001 for 60 μg/cm^2^). Post hoc analysis revealed significant differences between the sites indicated by the arrows and those summarized by their site code (in parentheses). In *B*, significant differences between sites classified by traffic and fraction are illustrated. NS, nonsignificant contrast. *p < 0.05.

We observed no IL-6 production in the supernatants of unexposed control cells. As with AA release, we observed the most marked increases in IL-6 following exposure to PM_2.5–10_, with more pronounced responses at the 60 μg/cm^2^ dose at most sites (Figure 2A). PM_0.1–2.5_ exposures did not significantly increase IL-6 production relative to controls at either concentration, with only MGH and HIA samples eliciting observable responses. IL-6 production in response to coarse PM exposure was significantly greater for samples from MGH (low traffic) compared with responses to samples from AMS (high traffic), DOR (moderate traffic), MOB (high traffic), and LYC (low traffic, wood burning) at 20 μg/cm^2^ and samples from MOB (high traffic) and LYC (low traffic) at 60 μg/cm^2^. We observed the greatest increase in IL-6 production after exposure to PM_2.5–10_ from the high-diesel-traffic HIA road tunnel site (mean ± SE, 972 ± 24 pg/mL), which was 34% of the maximal level induced by 1 μg/mL LPS (2,858 ± 152.98 pg/mL). As with AA, no differences were apparent in responses to samples grouped according to traffic intensity (Figure 2B). Responses to coarse PM samples were stronger than those observed with fine PM samples from comparable sites, but were significantly different only between samples from low-traffic sites.

### PM composition and associations with inflammatory responses

As we had a high degree of correlation between many of the 23 PAHs measured (data not shown), we grouped these compounds and analyzed them as low-molecular-weight (MW; < 190) and high-MW (> 190) species associated with PM_2.5–10_ and PM_2.5–10_, respectively. We observed the highest concentrations of both high- and low-MW PAHs at the rural Swedish (LYC) and the German high-traffic (MOB) sites, with clear differences in mean PAH concentrations according to MW and PM fraction across all sites [*p* < 0.001; see Supplemental Material, Figure 2 (doi:10.1289/ehp.1002105)]. PAH concentrations at the remaining high-traffic sites (HIA, ROM, and AMS) were comparable with those observed at the low-traffic sites (SAS and MGH). Neither AA release nor IL-6 production induced in response to PM was significantly correlated with concentrations of high- or low-MW PAHs in PM_2.5–10_ samples (see Supplemental Material, Table 1). In contrast, AA responses, but not IL-6 responses, were significantly correlated with PAH concentrations in PM_0.1–2.5_ samples [for low- and high-MW PAHs, respectively: individual sample means (*n* = 41), *r* = 0.489, *p* < 0.001 and *r* = 0.490, *p* < 0.001; site-specific mean concentrations (*n* = 9), *r* = 0.757, *p* < 0.05 and *r* = 0.757, *p* < 0.05], although associations were strongly influenced by the high PAH concentrations at LYC.

Concentrations of ionic species also varied markedly among the nine sites (NH_4_, *p* < 0.01; Cl^−^, *p* < 0.001; NO_3_, *p* = 0.01; SO_4_, *p* < 0.05), with evidence of higher concentrations at four of the five sampling locations in the Netherlands (excluding the HIA road tunnel) relative to the German, Italian, and Swedish sites [see Supplemental Material, Figure 3 (doi:10.1289/ehp.1002105)]. We noted no significant positive correlations between concentrations of any of the ionic species and AA and IL-6 responses to PM exposures, but we observed significant negative associations between PM_2.5–10_-induced AA release and NH_4_ and Cl^−^ content (see Supplemental Material, Table 2).

In the present analysis, we focused on PM elements associated with road dust resuspension [Fe, aluminum (Al), and silicon (Si)] (Viana et al. 2008), tire abrasion [zinc (Zn)] (Legret and Pagotto 1999), brake abrasion [Cu, barium (Ba), antimony (Sb)] (Boulter 2006; Garg et al. 2000; Sternbeck et al. 2002), and oil/fuel combustion processes, either as trace elements or fuel additives [manganese (Mn), lead (Pb), cerium (Ce), chromium (Cr), Ni, and V] (Park et al. 2008; Viana et al. 2008; for further details of sample composition, see Bloemen et al. 2005). Considering this restricted set of elements, two clear patterns were apparent across the nine sites, each characterized by strong internal correlations. The first pattern was associated with crustal elements [lithium (Li), Ce, Al, and Si], with the highest concentrations observed at high-traffic ROM, rural LYC, and the HIA road tunnel [see Supplemental Material, Figure 4 (doi:10.1289/ehp.1002105)]. The second pattern was associated with elements reflective of brake wear (Cu, Ba, and Sb), with the greatest concentrations observed in samples from the high-traffic sites (AMS, MOB, HIA, and ROM) and with total Fe showing a similar profile, especially with PM_2.5–10_ (see Supplemental Material, Figure 4). Of the elements considered, Fe, Cu, Cr, Mn, Sb, and Ba were positively associated with the AA response induced by PM_2.5–10_, both when the correlation was based on all 41 samples, and on the nine site-specific means (see Supplemental Material, Table 3). Significant associations with the AA response to PM_0.1–2.5_ were observed for Ba, Zn, and Pb only when all 41 samples were included in the analysis, and no consistent positive correlations were noted between elemental components and IL-6 responses to coarse or fine PM. Observed correlations were strongly influenced by the high metal concentrations in PM samples obtained from MOB (see Supplemental Material, Figure 5, using data for Cu as an example).

Endotoxin was present in all of the fine and coarse PM samples, with higher concentrations in PM_2.5–10_ samples and significant differences in site-specific mean PM_2.5–10_ endotoxin concentrations [*p* = 0.005; see Supplemental Material, Figure 6 (doi:10.1289/ehp.1002105)]. PM endotoxin content was not related to traffic density, with the highest concentrations observed at the high-traffic road tunnel (HIA) and the low-traffic Munich (MGH) background sites. PM_2.5–10_ endotoxin content was significantly correlated with IL-6 production (individual sample means: *r* = 0.56, *p* < 0.001; site-specific means: *r* = 0.92, *p* < 0.001).

### Inhibitor studies

**Figure 3:**
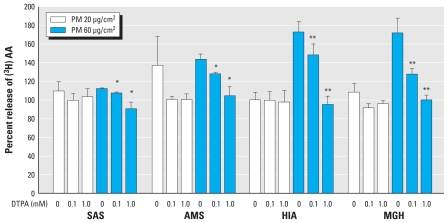
Effect of DTPA on PM-induced [^3^H]AA release from RAW264.7 cells labeled with [^3^H]AA for 1 hr and incubated for 5 hr with PM (20 or 60 μg/cm^2^), with or without pretreatment with DTPA (0.1 or 1 mM). [^3^H]AA release was measured in the supernatants and is expressed as the percentage of measured values in untreated cells; values are mean ± SE of four independent experiments measured in duplicate. *p < 0.05, and **p < 0.01 compared with corresponding samples without DTPA treatment.

We performed inhibitor studies on a subset of PM_2.5–10_ samples (chosen to reflect the full range of responses observed) to confirm whether statistical associations between PM metals and AA release and between PM endotoxin and IL-6 production were causal. Preincubation of coarse PM samples with the membrane-impermeable metal chelator DTPA at 0.1 and 1.0 mM abolished the AA response in a dose-dependent manner relative to the chelator-free controls (Figure 3). DTPA alone at the two indicated concentrations did not affect cell viability or AA release (data not shown).

**Figure 4:**
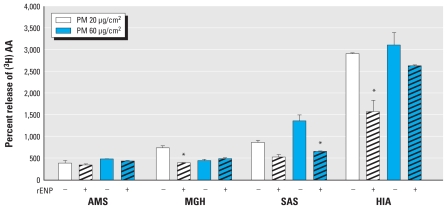
Effect of rENP on TNFα release from RAW264.7 cells exposed for 5 hr to ambient PM_0.1–2.5_ or PM_2.5–10_ from four different European sites at 20 μg/cm^2^ or 60 μg/cm^2^, with (hatched bars) or without (open bars) treatment with 2 μg/mL rENP. TNFα was partially but significantly inhibited by rENP in cells showing a high level of TNFα production. Bars represent means ± SE of three independent experiments assayed in duplicate. *p < 0.05 compared with corresponding samples without rENP treatment.

Because numerous PM samples, especially in the PM_0.1–2.5_ fraction, had failed to elicit any IL-6 response in the initial screening exercise, we investigated TNFα as an alternative cytokine to measure endotoxin-induced responses before conducting inhibition experiments with LPS-specific rENP. Specifically, we quantified IL-6 and TNFα after a 5-hr incubation with nine different PM_0.1–2.5_ and PM_2.5–10_ samples (at concentrations of 20 and 60 μg/cm^2^) from six sites [see Supplemental Material, Figure 7 (doi:10.1289/ehp.1002105)]. These data demonstrated strong correlations between 60 μg/cm^2^ PM_0.1–2.5_- and PM_2.5–10_-induced TNFα and IL-6 responses in RAW264.7 cells (PM_0.1–2.5_: *r* = 0.90, *p* < 0.001; PM_2.5–10_: *r* = 0.95, *p* < 0.001), with TNFα concentrations at measurable levels in all tested samples. Therefore, we used TNFα in the rENP inhibitor experiments. We observed significant partial reductions in TNFα responses following PM exposure in samples pretreated with rENP at both PM doses (Figure 4). Notably, whereas DTPA completely abolished AA responses to PM in all tested samples, incubation of the same samples with rENP elicited a variable degree of TNFα inhibition, with maximal and significant reductions observed with the SAS and HIA samples.

## Discussion

In this study we examined whether ambient PM_2.5–10_ and PM_0.1–2.5_ from high-traffic sites displayed enhanced proinflammatory properties consistent with evidence of increased respiratory symptoms in individuals with high exposures to traffic-related pollutants (Janssen et al. 2003). To address this question, we incubated RAW264.7 cells with PM samples from nine European sites with differing traffic contributions and characteristics and used the production of AA, IL-6, and TNFα as output measures of PM inflammatory potential.

We chose macrophages for this screening exercise because of their central role in the coordination of the inflammatory response (Becker et al. 2005; Ghio and Devlin 2001) and extensive evidence of their involvement in PM-induced responses in animal models (Kodavanti et al. 2002; Wegesser et al. 2009) and human volunteers (Ghio and Devlin 2001; Schaumann et al. 2004). Macrophages also play a critical role in the effector arm of the immune response via phagocytosis of foreign material, which results in production of ROS and release of lysosomal enzymes. Under normal situations these functions are beneficial, but aberrant activation of these “killing” mechanisms can result in injury to healthy tissue, resulting in further oxidative stress and amplification of the inflammatory response (Ayres et al. 2008; Prahalad et al. 2001; Xia et al. 2006) *in vivo*.

Our focus on the macrophagic response means that our end points can be viewed only as a proxy measure of PM inflammatory potential; a more comprehensive measure would require evaluation of a broader array of relevant cells types at the air–lung interface. We selected the 5-hr time point to assess effects based on our previous observations with RAW264.7 cells challenged with ambient PM and LPS (Guastadisegni et al. 1997; Pozzi et al. 2005), but our results should be interpreted with caution because this single exposure duration may not be ideal for assessing potential effects on all end points.

We examined the capacity of PM to stimulate AA release or IL-6 production using all coarse and fine PM samples from each of the nine sites. Importantly, although AA and IL-6 both are potent proinflammatory mediators, with AA acting as a substrate for the downstream synthesis of prostaglandins and leukotrienes (via cyclooxygenase and 5-lypoxygenase pathways, respectively), we did not observe quantitative associations between responses based on these two markers. This further highlights the difficulty in deriving a simple measure of PM inflammatory potential, because the induction of separate pathways can elicit a common final inflammatory response. Nevertheless, the following observations were clear from the initial screening exercise. First, the relative potential for PM to elicit the release of proinflammatory mediators from RAW264.7 cells varied among samples obtained from different sites when examined on an equivalent concentration basis. Second, we found no clear evidence that AA or IL-6 responses were greater at high- versus low-traffic sites. Indeed, samples from several of the low-traffic sites elicited some of the largest responses we observed: LYC for AA and MOB for IL-6. Third, neither dose of PM elicited any measurable cytotoxicity. Fourth, the site contrasts in PM induction were more marked for IL-6 production than for AA release. Finally, AA and IL-6 responses to PM_2.5–10_ fractions were generally stronger than those observed in response to parallel PM_0.1–2.5_ samples.

These observations do not support our original hypothesis that PM from high-traffic environments would display an enhanced capacity to trigger inflammation, which had been found previously *in vivo* in spontaneously hypertensive rats (Gerlofs-Nijland et al. 2007). However, responses observed at equivalent exposure doses (during the experimental incubations) are scalable to the concentration of PM in the ambient air at each of the sampled locations. Hence, although responses to PM samples collected at LYC (low traffic, wood burning), MGH (low traffic), and AMS (high traffic) sites were comparable when exposures were on an equal mass basis, in reality one would expect greater inflammatory responses in the lungs of subjects breathing air at the roadside sites with higher ambient PM concentrations. For example, the estimated annual average PM_10_ concentration at AMS was 27.7 μg/m^3^, compared with 17.9 μg/m^3^ at MGH (Janssen et al. 2008). Consequently, when we compare effects of PM on an equivalent mass basis, we are investigating the contribution of relative source-specific components of PM to the observed response. Importantly, we did not evaluate ultrafine PM fractions (PM_< 0.1_) that are known to be elevated at roadside microenvironments (McCreanor et al. 2007) and may be highly significant in terms of potential health impacts (Knol et al. 2009).

To explore the influence of specific sources, we estimated associations between the magnitudes of responses and the composition of the PM from each of the sites. For the AA response, particularly that elicited by the PM_2.5–10_ fraction, we observed significant associations with elements characteristic of mechanical abrasion processes: both brake (Cu, Sb, and Ba; Garg et al. 2000; Boulter 2006; Schauer et al. 2006) and generic engine (Fe and Mn; Schauer et al. 2006) wear. Similar associations were not apparent with the same elements in PM_0.1–2.5_, consistent with a general absence of responses to fine PM from all sites, with the exception of LYC and SAS (both low traffic). Notably, increased inflammatory responses after exposure to fine PM from LYC have also been observed after intratracheal instillation into the lungs of spontaneously hypertensive rats (Gerlofs-Nijland et al. 2007). Responses to PM_2.5–10_ were not correlated with any of the organic (PAH) markers employed and were inversely associated with NH_4_ and Cl^–^ concentrations. This latter observation suggests that an enrichment of these secondary species in the PM samples was actually diluting the contribution of other active constituents, implying that they themselves had minimal biological potency. AA release was increased in response to fine PM from a few low-traffic sites only, but was correlated with both low- and high-MW PAH concentrations. This association was strongly influenced by the AA response to PM samples from LYC, a low-traffic site with significant contributions from wood burning that was highly enriched with PAHs relative to all other sites.

Although correlation analyses are useful for exploring potential determinants of PM-induced responses, it is not possible to rule out effects of other unmeasured toxic components derived from the same source. To demonstrate causation, it is therefore necessary to establish whether removing a given component inhibits the response. Of the elements that were positively associated with AA responses, both Cu and Fe are known redox catalysts, capable of generating ROS *in vivo* (Valko et al. 2005). Because oxidative stress triggers the release of inflammatory mediators (Ayres et al. 2008; Xia et al. 2006), including AA (Pawliczak et al. 2002), there is a plausible basis for believing these metals may play a role in inducing the AA response. To confirm this, we pretreated PM with the metal chelator DTPA before incubation with the cell line to bind and catalytically inactivate both Fe and Cu (Fisher et al. 2004). DTPA treatment at 1 mM effectively abolished the AA responses observed with exposure to a selected panel of coarse PM, consistent with a causal role of Cu and Fe in the response. However, it is not clear whether this reflected effects of extracellular ROS generation in the incubation media or the induction of intracellular oxidative stress following PM uptake.

In contrast to the AA response, IL-6 production appeared to be unrelated to metal or organic PM components. However, we observed a strong correlation between the endotoxin content of coarse PM and the IL-6 response. Consistent with other published studies (Ning et al. 2000; Osornio-Vargas et al. 2003; Pozzi et al. 2005), we observed higher endotoxin concentrations in PM_2.5–10_ than in PM_0.1–2.5_. Endotoxin is a cell wall polymer present in gram-negative bacteria that has been shown to stimulate macrophages through binding to the CD-14/MD-2/Toll-like receptor to trigger a variety of signaling pathways that result in IL-6 and TNFα release (Becker et al. 2005; Monn and Becker 1999). These two cytokines have also been shown to be elevated in response to both PM (Wang et al. 2008) and LPS (Hoogerwerf et al. 2008) challenges, providing a plausible basis for an effect of endotoxin on the IL-6 response. To investigate this further, we compared associations between TNFα release and PM endotoxin concentrations in the presence and absence of rENP, which detoxifies endotoxin (Fletcher et al. 1993), after demonstrating strong correlations between TNFα and IL-6 responses. In contrast to the complete inhibition of the AA response by DTPA, coincubation of the PM samples with rENP elicited only a partial blunting of PM-induced TNFα release, suggesting that other factors (e.g., other biological materials derived from gram-positive bacteria or fungi) may be contributing to this response.

It was also notable that the coarse PM fraction appeared more active in the assays we performed than the fine PM, particularly for coarse PM from high-traffic sites for AA release and from low-traffic sites for IL-6. This observation is consistent with several recent *in vitro* studies (Dybing et al. 2004; Hetland et al. 2005; Jalava et al. 2007) and with an *in vivo* study of intratracheal exposures that indicated that the coarse PM fraction has significant biological activity (Gerlofs-Nijland et al. 2007). Clearly, enhanced activity of coarse PM in an *in vitro* assay, and with exposure by intratracheal instillation *in vivo*, does not necessarily imply that comparable effects would be observed in response to inhaled PM, because PM deposition within the respiratory tract would be expected to affect the magnitude of inflammatory responses to different size fractions *in vivo*. However, these observations do suggest that the larger PM fractions are potentially toxic and should not be ignored during a move toward regulation of PM_2.5_, a view supported by recent epidemiological evaluations of the short-term health effects of coarse PM (Brunekreef and Forsberg 2005; Lin et al. 2002).

## Conclusions

We investigated variation in the proinflammatory potential of ambient fine and coarse PM collected at sites with varying source (traffic, wood burning) contributions by examining AA release and IL-6/TNFα production in response to exposure in a murine macrophagic cell line. We hypothesized that PM from high-traffic sites would display the greatest reactivity, consistent with increased respiratory symptoms observed in populations living in close proximity to high-traffic roads. PM samples from the high-traffic sites were enriched with metals related to tire and break abrasion, but we observed no simplistic relationship between traffic intensity and the PM-induced inflammatory cellular response. Although the traffic-related components were correlated with the release of the prostanoid precursor AA, many of the more active PM samples were obtained from the low-traffic sites. This may have been partly related to site-specific differences in endotoxin concentrations, which appeared to contribute to the IL-6/TNFα response, and may contribute significantly to the potential inflammatory burden of the PM airshed. Our findings also demonstrated that PM_2.5–10_ from some sites had greater inflammatory potential than PM_0.1–2.5_ from the same sites, emphasizing the need to continue regulating PM in this size range.
